# Insights into the Roles of Midazolam in Cancer Therapy

**DOI:** 10.1155/2017/3826506

**Published:** 2017-06-19

**Authors:** Jinghua Jiao, Yuheng Wang, Xiaofeng Sun, Xiaojing Jiang

**Affiliations:** ^1^Department of Anesthesiology, The First Affiliated Hospital, China Medical University, Shenyang 110001, China; ^2^Department of Anesthesiology, Central Hospital, Shenyang Medical College, Shenyang 110024, China

## Abstract

With its high worldwide mortality and morbidity, cancer has gained increasing attention and novel anticancer drugs have become the focus for cancer research. Recently, studies have shown that most anesthetic agents can influence the activity of tumor cells. Midazolam is a *γ*-aminobutyric acid A (GABA_A_) receptor agonist, used widely for preoperative sedation and as an adjuvant during neuraxial blockade. Some studies have indicated the potential for midazolam as a novel therapeutic cancer drug; however, the mechanism by which midazolam affects cancer cells needs to be clarified. This systematic review aims to summarize the progress in assessing the molecular mechanism of midazolam as an anticancer agent.

## 1. Introduction

Cancer has become the most common disease worldwide and is the leading cause of death [1]. Currently, the primary treatment for a solid tumor is still surgical resection. During surgery, anesthesia and the drugs used may affect the tumor and result in the release of tumor cells into the blood, lymphatic system, bone marrow, and even organs, leading to the formation of micrometastatic lesions, an increased risk of tumor recurrence and metastasis, and ultimately affecting postoperative survival rate [2, 3]. A number of in vitro studies have confirmed that most anesthetic agents, including midazolam, have substantial antitumor effects [4–6]. To date, a few studies have used various cell lines to determine the mechanism underlying the effect of midazolam on cancer cells. However, this mechanism is multifaceted and the means by which midazolam affects a variety of cancer signaling pathways needs to be clarified. This article reviews the biochemical properties of midazolam, its activity, its antitumor properties, and the possible mechanisms involved. We hope to provide a theoretical basis for the potential clinical application of midazolam as a therapeutic agent for tumors.

## 2. Chemistry and Clinical Pharmacology of Midazolam

**Figure 1:**
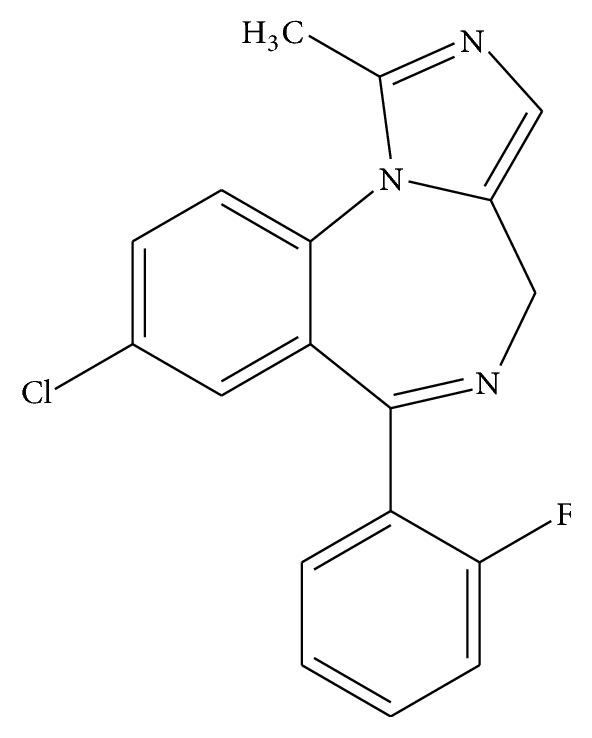
The chemical structure of midazolam (dormicum).

The chemical structure of midazolam (dormicum) is 8-chloro-6-(2-fluorophenyl)-1-methyl-4H-imidazo[1,5-a][1,4]benzodiazepine (Figure 1).

As a benzodiazepine, anticonvulsant drug, midazolam has a rapid onset and short-lasting effect. In addition, midazolam has significant hypnotic, anxiolytic, amnesic, and sedative properties and these occur via modulation of the GABA_A_ receptor in the central nervous system [7, 8]. In the clinical situation, midazolam is the current drug of choice for sedation, including preoperative sedation. For patients undergoing caesarean section under spinal anesthesia, midazolam is effective for the prevention of nausea and vomiting [9] and produces postoperative pain relief [10].

However, midazolam was recently reported to be among the 20 most often utilized medications in cancer patients to be associated with toxic side effects [11]. In addition, it has been shown to have neuronal cytotoxicity and apoptosis-inducing activity in hematogenic, ectodermal, mesenchymal, and neuronal cells [12–15].

## 3. Systematic Review

### 3.1. Search Strategy

A systematic and comprehensive literature search was performed by two authors (J.J.H. and J.X.J.) independently and using the PubMed, Embase, Web of Science, Ovid evidence-based medicine, Chinese science and technology periodicals (CNKI, VIP, and Wan Fang), and Chinese Biomedical Literature (CBM) databases, for publications up to March 5, 2017. Disagreement between reviewers was resolved by discussion. The following terms were used in each search: cancer, carcinoma, neoplasm, tumor, midazolam, dormicum, and 8-chloro-6-(2-fluorophenyl)-1-methyl-4H-imidazo[1,5-a][1,4]benzodiazepine. The results were limited to English or Chinese language.

### 3.2. Inclusion and Exclusion Criteria

Any study that clearly stated a link between midazolam and cancer, both in vitro and in vivo, was included. Studies were excluded based on the following criteria: (1) conference abstracts, reviews, conference papers, case reports, editorials, comments, news, congresses, and letters; (2) non-English or Chinese.

### 3.3. Quality Assessment and Data Extraction

The quality of all eligible studies was assessed by two independent reviewers using the EBLIP Critical Appraisal Checklist [16]. The extracted data from included studies are shown in Table 1.

## 4. Results and Discussion

**Figure 2:**
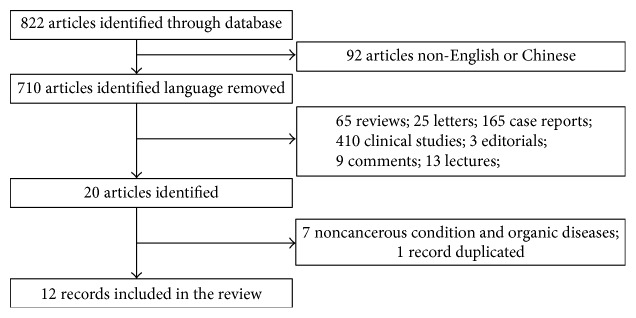
Flow diagram of the studies identification and selection.

A total of 822 studies were identified in PubMed, Embase, Web of Science, Ovid evidence-based medicine, Chinese science and technology periodicals (CNKI, VIP, and Wan Fang), and Chinese Biomedical Literature (CBM) using our search strategy. After detailed screening, 12 studies were considered eligible for inclusion in this review (summarized in Figure 2).

### 4.1. Possible Antitumor Mechanisms of Midazolam In Vitro

There are thirty-three trillion cells in the human body and numerous cellular functions that maintain the balance of these cells. In disease situations, this balance may be disrupted by multiple external stimuli, stress, and the generation of mutant cells [17]. Cell death plays an important role in maintaining cellular balance by removing cells that are “unnecessary” or potentially harmful [18] and may occur via two means, necrosis and apoptosis. Apoptosis is a programmed cell death, whereas necrosis is an “accidental” death resulting from a physical or chemical assault [19]. During the process of necrosis, the cell membrane is distorted and the cell nucleus disintegrates resulting in degradation products. Apoptosis, on the other hand, is a far more regulated process that results in the cell degenerating into contained apoptotic bodies that can be phagocytosed and removed.

### 4.2. Induction of Apoptosis

Apoptosis plays a crucial role in eliminating cells that are unnecessary or harmful and it also has a role in numerous biological processes, including cell differentiation and proliferation [20, 21]. With respect to cancer, apoptosis has become a popular target for many treatment strategies as there is a close relationship with apoptosis and many of the processes involved in cancer progression [22–27].

Previous studies using flow cytometry showed that midazolam induced apoptosis in the human lymphoma and neuroblastoma cell lines, MA-10 Leydig tumor cells, and the mantle cell lymphoma cell line, JeKo-1, in a concentration-dependent manner [12, 29, 37]. Potential biomarkers of apoptosis include B cell lymphoma 2 (Bcl-2) family proteins, caspase-3, and caspase-9. In the mantle cell lymphoma cell line, JeKo-1, a dose-dependent reduction of Bcl-2, procaspase-9 and procaspase-3 protein expression and an increase in cyto-C protein expression were found. The expression of procaspase-8 protein did not change. It was concluded that midazolam potentially initiates the mitochondrial pathway, not the death receptor pathway, by reducing the expression of Bcl-2, leading in turn to the release of cyto-C in mitochondria. This leads to the activation of caspase 9 and caspase 3 protein and triggers the caspase cascade, ultimately leading to the induction of apoptosis in the JeKo-1 cells [37].

However, in the human lymphoma and neuroblastoma cell lines, Bcl-2 overexpression and caspase 9 deficiency protected against midazolam toxicity, whereas a deficiency in caspase 8 or Fas-associating protein with a novel death domain (FADD) had no effect. Although pancaspase inhibition had a strong protective effect, flumazenil could not inhibit midazolam-induced apoptosis. Midazolam induced apoptosis via activation of the mitochondrial pathway in a concentration-dependent manner. The induction of apoptosis by midazolam is presumably unrelated to GABA_A_ receptor pathway signaling [12].

The endoplasmic reticulum stress (ER stress) pathway, also known as the Unfolded Protein Response, is the response of the cell to a dangerous buildup of unfolded or misfolded proteins in the ER [39–41]. C/EBP-homologous protein (CHOP), activating transcription factor 4 (ATF-4), and phosphorylated *α* subunit of eukaryotic initiation factor 2 (p-eIF2*α*) are typical ER stress markers [42]. eIF2*α*/ATF4/CHOP is an essential signal pathway regulating ER stress [43]. In MA-10 Leydig tumor cells, where apoptosis is induced with midazolam, the overexpression of p-eIF2*α*, ATF4, ATF3, and CHOP was observed, suggesting that midazolam may induce apoptosis via the ER stress pathway [31]. Midazolam was also suggested to induce the activation of caspase-8, caspase-9, and caspase-3 and poly(ADP-ribose) polymerase proteins in the mouse Leydig tumor cells. There were no changes in the levels of Bcl-2 associated X protein (Bax) (a proapoptotic family member) [44], but Bid (also proapoptotic and which is activated by various death stimuli) was significantly decreased after midazolam intervention [45, 46]. Midazolam decreases the expression of pAkt and Akt and upregulates the phosphorylation of p38 and c-Jun NH2-terminal kinase, rather than extracellular signal-regulated kinases [29]. Thus, midazolam-induced apoptosis may be induced via the activation of the caspase cascade, the inhibition of the pAkt pathway, and the induction of p38 and c-Jun NH2-terminal kinase pathways [29].

### 4.3. Necrosis

In decreasing order, midazolam showed the greatest toxicity for HL-60 cells, epidermal keratinocytes, oral squamous cell carcinoma (OSCC), and glioblastoma cells. Midazolam did not induce the generation of apoptosis markers in OSCC cells (including DNA cleavage between nucleosomes and activation of caspase-3, caspase-8, and caspase-9) but did induce many vacuoles, mitochondrial swelling, and cell membrane rupture [32]. Midazolam cytotoxicity for human lymphoma and neuroblastoma cell lines affected a switch from caspase-dependent apoptosis to necrosis as the concentration increased [12].

### 4.4. Autophagy

Autophagy is a newly recognized innate defense mechanism that has been observed in cancer and is a physiological program that enables the body to deal with the destruction of cells [47]. Autophagy maintains a homeostatic balance via protein degradation and the turnover of destroyed cellular organelles [48]. Autophagy was not induced with midazolam as midazolam cytotoxicity was not reduced by pretreatment with autophagy inhibitors (3-methyladenine and bafilomycin A1) in the OSCC cell lines [30]. Thus, midazolam appears to induce necrosis, and not apoptosis or autophagy, in OSCC cell lines.

However, in MA-10 cells, the staining and expression of LC3-phosphatidylethanolamine conjugate (LC3-II), which is recruited to autophagosomal membranes, was observed following midazolam treatment, suggesting that midazolam induced autophagy in MA-10 cells [31].

### 4.5. Effect on the Cell Cycle

The rate of cell proliferation is always determined by cell cycle distribution. There are four phases in the cell cycle including G1, G2, S, and M; G1 and G2 phases are gap phases; S phase is the synthesis phase during which the genetic material is duplicated; and the M phase is where mitosis partitions the genetic material and the cell divides [49, 50]. As the genetic material is duplicated in the S phase, the percentage of cells in this phase also reflects the rate of proliferating cells. As the balance between proliferation and apoptosis is destroyed in tumor cells, the percentage of tumor cells in the S phase is much larger than that in normal cells from the same tissues or organs [51].

Another study that looked at the effect of midazolam in mouse Leydig tumor cells showed an accumulation of MA-10 cells in the sub-G_1_ phase and a reduction of cells in the G_2_/M phase in a time- and dose-dependent manner [29]. It was suggested that midazolam may inhibit the expression of cyclin-A, cyclin-B, and cyclin-dependent kinase 1 (CDK1) in MA-10 cells and alter the phosphorylation of P21, P27, and p53, thus controlling the cell cycle through the regulation of the p53 pathway [31].

On the other hand, a study by Dou and coworkers showed that midazolam triggered G_0_/G_1_ cell cycle arrest in the human head and neck squamous carcinoma FaDu cell line by regulating cell cycle regulators [34].

### 4.6. Inhibition of Proliferation

The human E1A binding protein, p300, also known as EP300 or p300, is encoded by the EP300 gene and regulates the activity of many genes in tissues throughout the body [52]. It plays an essential role in regulating cell growth and division, prompting cells to mature, differentiate, and assume specialized functions, and preventing the growth of cancerous tumors [53]. The p300 protein appears to be critical for normal development before and after birth. The p300 protein carries out its function by activating transcription, the process of translating the genetic blueprint into protein production. Specifically, p300 connects transcription factors and proteins that initiate the transcription process with numerous proteins that carry out the transcription process in the cell's nucleus [54].

Midazolam was shown to inhibit the proliferation of FaDu cells, a cell line from a squamous cell carcinoma of the hypopharynx, and attenuated the mRNA and protein levels of p300. The knockdown of p300 resulted in an upregulation of p21 and p27 proteins and downregulation of p-Rb protein. Thus, it appeared that midazolam inhibited the proliferation of FaDu cells via downregulation of p300 expression [33].

Similarly, another study on FaDu cells showed that midazolam was able to inhibit the growth and proliferation of FaDu cells [34]. However, this study reported that the inhibition of FaDu cell proliferation was mediated by the targeting of transient receptor potential melastatin 7 (TRPM7). TRPM7, which is expressed in human head and neck squamous carcinoma cells, is one of the TRP channel family members. The growth and proliferation of FaDu cell lines can be inhibited by the inhibition of TRPM7 expression or blocking of TRPM7 channels [55]. Similar results were found in a malignant glioblastoma cell line, T98-MG cell [36].

A further study demonstrated that midazolam inhibited the growth and proliferation of SW480 colonic adenocarcinoma cells in a time- and dose-dependent manner and downregulated ubiquitin-specific protease 22 (USP22) expression. With the use of USP22 small interfering RNA (Si-RNA), they were able to silence USP22 expression and found that SW480 cell proliferation was inhibited, while P21 and P27 expression was upregulated, and pRB downregulated. Thus, a feasible mechanism by which midazolam inhibits proliferation may be via the mediation of cyclin-dependent kinase inhibitor/retinoblastoma protein (CDKI/RB) pathways through the downregulation of USP22 [38].

Midazolam has been shown to inhibit the in vitro growth and differentiation of two murine myeloid leukemia cell lines (WEHI 3B (JCS) and M1 cells) in a dose-dependent manner [28]. Midazolam enhanced the expression of the differentiation antigens Mac-1, F4/80, and Gr-1 in the cells and expression of tumor necrosis factor (TNF-alpha), and the neutrophil-specific J11d differentiation marker was significantly upregulated in midazolam-treated JCS cells.

### 4.7. Antitumor Effects of Midazolam in Animal Models and Possible Mechanisms

There has only been one animal model study. This was carried out in BALB/c-nu mice bearing K562 and HT29 cell human tumor xenografts. The results showed that midazolam inhibited growth of the cancer cells via activation of the mitochondrial intrinsic pathway of apoptosis and inhibited HT29 tumor growth in the xenograft mice. The mechanism of inhibition of carcinogenesis by midazolam may be a suppression of reactive oxygen species (ROS) production leading to modulation of apoptosis and growth regulatory proteins [35].

## 5. Concluding Remarks

It is interesting to consider the possibility that, besides its use as an anesthetic agent, midazolam may have the ability to prevent or inhibit tumor development. New insights are rapidly being gained into the role of the midazolam in cancer treatment. In this review, the protective role of midazolam in cancer and the potential mechanisms underlying this have been described. Studies suggest a critical role for midazolam in influencing many signaling pathways on which cancer cells death is induced including necrosis and apoptosis. Midazolam can also inhibit the proliferation of cancer by inhibiting cell cycle progression. However, the impact of midazolam on other behavior of tumor cells, such as invasion and metastasis, remains to be further studied.

In relating observations in vitro to molecular events in vivo, a main focus is the different concentrations of midazolam used. In vitro studies, the concentrations of midazolam were usually from 0 to 100 *μ*M, even achieving 200 *μ*M or 1000 *μ*M. The effect of different concentrations of midazolam on tumor cells was shown in Table 1. However, only one study has sought to evaluate, in vivo, the effect of midazolam on cancer preventive activities and the concentration of midazolam was 0.83 mg/kg body weight for BALB/c-nu mice bearing K562 and HT29 cells human tumor xenografts [35]. Further in vivo studies are needed to evaluate the effect of midazolam on cancer preventive activities and even address the relationship between the effective concentrations of midazolam in vivo versus in vitro.

The above discussions on the anticancer activities of midazolam are based mostly on studies with lymphoma cells and Leydig tumor cells. These theories will serve as a basis for researchers to explore the effect of midazolam on other tumors. For example, we are investigating the effect of midazolam on lung cancer cells (A549), and we found that midazolam could induce the apoptosis of A549 cells through regulating signal transducer and activator of transcription 3 (Stat3) signaling pathway. Although the detailed mechanism by which midazolam acts on cancer cells remains elusive, the results described above suggest that midazolam could present a potential therapeutic in various cancers. Further study into the role of midazolam in the prevention of cancer is crucial if translation from the laboratory to the clinical setting can occur.

## Figures and Tables

**Table 1:** Characteristics of included studies.

First author	Year	Area	Study design	Cell lines/animal	Concentrations of midazolam	Pathway	Assays	Results	Conclusion
Stevens [12]	2011	Netherlands	In vitro	Human lymphoma and neuroblastoma cell lines	0, 50, 70, 100, and 150 *μ*M for Jurkat T-lymphoma cells 0, 100, 200, 300, and 400 *μ*M neuroblastoma cells	Mitochondrial pathway	XTT assay	Midazolam induced apoptosis in all investigated cell types in a concentration-dependent manner. Bcl2 overexpression and caspase-9 deficiency protected against toxicity, whereas caspase-8 or FADD deficiency had no effect.	Midazolam induces apoptosis via activation of the mitochondrial pathway in a concentration-dependent manner. The mechanism of midazolam toxicity switches from caspase-dependent apoptosis to necrosis with increasing concentrations of midazolam.

Mak [28]	1997	Hong Kong	In vitro	Murine myeloid leukemia WEHI 3B (JCS) and M1 cells	0, 10, 20, 30, and 40 *μ*g/ml for both JCS and M1 cells for 18, 48, and 72 hours	NA	Flow cytometry; mRNA phenotyping; phagocytosis; cell morphology; Southern-blot	Midazolam inhibits proliferation of both M1 and JCS cells in a dose-dependent manner. mRNA phenotyping also indicated that the expression of tumor necrosis factor and the neutrophil-specific J11d differentiation marker was significantly upregulated in midazolam-treated JCS cells. In addition, the phagocytic activity of midazolam-treated JCS cells was increased towards opsonized yeast cells.	Midazolam inhibits proliferation of both M1 and JCS cells.

So [29]	2014	Taiwan	In vitro	MA-10 mouse Leydig tumor cell line	0, 6, 30, and 150 *μ*M for 3, 6, 12, and 24 hours	pAkt pathway and p38 and c-Jun NH2-terminal kinase pathways	Flow cytometry and immunoblotting analysis	Midazolam induced the accumulation of the MA-10 cell population in the sub-G1 phase and a reduction in the G2/M phase, in a time- and dose-dependent manner. It induced the activation of caspase-8, caspase-9, and caspase-3 and poly(ADP-ribose) polymerase, decreased both pAkt and Akt expression, and stimulated the phosphorylation of p38 and c-Jun NH2-terminal kinase.	Midazolam induced MA-10 cell apoptosis via activation of the caspase cascade, the inhibition of pAkt pathway, and the induction of p38 and c-Jun NH2-terminal kinase pathways.

Ohno [30]	2012	Japan	In vitro	Human OSCC cell lines; HL-60 cells; glioblastoma; keratinocytes; oral normal cells	0–1000 *μ*M for 3, 6, 9, 12, 24, and 48 hours	NA	Cytotoxic activity by MTT; DNA fragmentation by ultraviolet irradiation; caspase activation; autophagy; electron microscopy	Midazolam showed the highest cytotoxicity. In HL-60 cells, it induced the appearance of many vacuoles, mitochondrial swelling, and cell membrane rupture in HSC-2 and HSC-4 cells.	Midazolam may induce necrotic cell death, rather than apoptosis or autophagy, in OSCC cell lines.

So [31]	2016	Taiwan	In vitro	MA-10 mouse Leydig tumor cells	0, 6, 30, and 150 *μ*M for 1, 3, 6, 12, and 24 hours	ER stress and p53 pathway	Flow cytometry assay and Western blot analyses	Midazolam significantly decreased cell viability but increased sub-G1 phase cell numbers and apoptosis. Expression of Fas, Fas ligand, p-EIF2*α*, ATF4, ATF3, CHOP, and LC3-II proteins was detected. Midazolam was able to regulate the cell cycle via regulation of the p53 pathway.	Midazolam induced cell apoptosis in MA-10 mouse Leydig tumor cells through activation of ER stress and regulation of the cell cycle via the p53 pathway, with the involvement of autophagy.

Braun [32]	2015	Germany	In vitro	Human neuroblastoma cells	2, 4, 8, 16, 128, 256, and 512 *μ*M for 24 and 48 hours	NA	Cell proliferation; cell cycle; cell viability	Midazolam increased cell viability at lower concentrations, whereas higher concentrations reduced cell viability.	Midazolam causes a hormetic dose-response relationship in human neuroblastoma cells.

Dou [33]	2014	China	In vitro	Hypopharyngeal squamous carcinoma cells	0, 6.25, 12.5, 25, 50, and 100 *μ*M for 24 and 48 hours	CDKI/RB pathway	MTT and BrdU incorporation RT-PCR and Western blotting	Midazolam inhibited the expression of p300 and the proliferation of FaDu cells. Additionally, knockdown of p300 resulted in increased expression of p21 and p27 and decreased expression of p-Rb, while inhibiting the proliferation of FaDu cells.	Midazolam inhibits the proliferation of human head and neck squamous carcinoma cells by downregulating p300.

Dou [34]	2013	China	In vitro	FaDu human hypopharyngeal squamous cell carcinoma cells	0, 6.25, 12.5, 25, 50, and 100 *μ*M for 24 and 48 hours	TRPM7 inhibition	Cell death assay; cell cycle analysis; Western blot analysis; quantitative real-time PCR	Midazolam inhibits the growth and proliferation of FaDu cells. Midazolam triggers G0/G1 cell cycle arrest by regulating cell cycle regulators. The inhibitory effect of midazolam on proliferation is benzodiazepine receptor- (BR-) independent but TRPM7-dependent.	The inhibitory activity of midazolam on cancer cell growth and proliferation, combined with the TRPM-dependent mechanism, reveals the anticancer potential of midazolam as a TRPM7 inhibitor.

Mishra [35]	2013	Korea	In vitro; in vivo	BALB/c-nu mice bearing K562 and HT29 cells human tumor xenografts	0, 10, 30, 100, and 200 *μ*M for 24 and 48 hours (in vitro), 0.83 mg/kg body weight (in vivo)	Mitochondrial intrinsic pathway	Cell viability; flow cytometric analysis; cell cycle analysis; measurement of mitochondrial membrane potential; DNA fragmentation; Western blot; intracellular superoxide generation; in vivo mouse xenograft	Midazolam decreased the viability of K562 and HT29 cells by inducing apoptosis and S phase cell-cycle arrest in a concentration-dependent manner. Midazolam activated caspase-9, caspase-3 and PARP, lowered mitochondrial membrane potential, increased apoptotic DNA fragmentation, and exhibited ROS scavenging activity through the inhibition of NADPH oxidase 2 (Nox2) enzyme activity in K562 cells. It also resulted in inhibition of pERK1/2 signaling which led to inhibition of the antiapoptotic proteins Bcl-XL and XIAP and phosphorylation activation of the proapoptotic protein Bid. Midazolam inhibited growth of HT29 tumors in xenograft mice.	Midazolam inhibited the growth of cancer cells via activation of the mitochondrial intrinsic pathway of apoptosis. It also inhibited HT29 tumor growth in xenograft mice. The mechanism underlying these effects may be suppression of ROS production leading to modulation of apoptosis and growth regulatory proteins.

Chen [36]	2016	China	In vitro	Malignant glioblastoma cell line T98-MG cells	0, 25, 50, and 100 *μ*M for 24 and 48 hours	Inhibited the TRPM7 expression	Immunofluorescence; cell proliferation and cell viability; caspase-3 activity and LDH release assay; reverse transcription-PCR; electrophysiology; cell cycle analysis; Western blot analysis; calcium imaging	Brief midazolam treatment (seconds) suppressed TRPM7 channels and calcium influx, while treatment for 48 h inhibited the TRPM7 expression. The inhibitory effect on TRPM7 accounts for the decrease in proliferation and G0/G1 phase cell cycle arrest induced by midazolam.	Midazolam represses proliferation of human malignant glioma cells via the inhibition of TRPM7 channels.

Hong [37]	2013	China	In vitro	Mantle cell; lymphoma JeKo-1 cell line	10, 20, 40, and 80 *μ*mol/L for 24, 48, 72 hours	Mitochondrial pathway	CCK8; flow cytometry; Western blot	Midazolam inhibited the growth of JeKo-1 cells; it induced apoptosis and reduction of Bcl-2, procaspase-9, and procaspase-3 protein expression and an increase in cyto-C protein expression in a concentration-dependent manner.	Midazolam may initiate the mitochondrial pathway by reducing the expression of Bcl-2, leading in turn to the release of Cyto-C in the mitochondria. This leads to the activation of caspase-9 and caspase-3 protein and triggers the caspase cascade, ultimately inducing apoptosis of the JeKo-1 cells.

Dou [38]	2012	China	In vitro	Human colon cancer SW480 cells	0, 6.25, 12.5, 25, 50, and 100 *μ*M for 24 and 48 hours	CDKI/Rb pathway	MTT and BrdU incorporation RT-PCR and Western blotting	Midazolam inhibited the growth and proliferation of SW480 cells in a time- and dose-dependent manner. It downregulated USP22 expression. When USP22 expression was silenced by siRNA, proliferation of the SW480 cells was inhibited. P21 and P27 expression was upregulated, while pRB was downregulated.	Midazolam inhibits the proliferation of human colon cancer SW480 cells. The mechanism may be through mediation of the CDKI/RB pathway via downregulation of USP22.

GABAA, *γ*-aminobutyric acid A; CBM, Chinese Biomedical Literature; Bci-2, B cell lymphoma 2; FADD, Fas-associating protein with a novel death domain; ER stress, endoplasmic reticulum stress; CHOP, C/EBP-homologous protein; ATF-4, activating transcription factor 4; p-eIF2*α*, phosphorylated *α* subunit of eukaryotic initiation factor 2; Bax, Bcl-2 associated X; OSCC, oral squamous cell carcinoma; LC3-II, LC3-phosphatidylethanolamine conjugate; CKD1, cyclin-dependent kinase 1; TRPM7, transient receptor potential melastatin 7; USP22, ubiquitin-specific protease 22; CDKI/RB, cyclin-dependent kinase inhibitor/retinoblastoma protein; TNF, tumor necrosis factor; ROS, reactive oxygen species; NA: not applicable.
